# How to talk about genome editing

**DOI:** 10.1093/bmb/ldy015

**Published:** 2018-04-25

**Authors:** Sandy Starr

**Affiliations:** Progress Educational Trust, 140 Grays Inn Road, London, UK

**Keywords:** genome editing, public understanding, DNA, CRISPR, fertility, embryo

## Abstract

**Background:**

Human genome editing is an area of growing prominence, with many potential therapeutic applications.

**Sources of data:**

A project by two UK charities, whose participants included fertility sector patients and practitioners and also people affected by genetic disease and rare disease. Scientific research into, and wider discussion of, genomics and genome editing.

**Areas of agreement:**

There is a need for improved public and professional understanding of genome editing.

**Areas of controversy:**

The way genome editing is discussed is often inconsistent and confusing. Simply defining and explaining the term ‘genome’ can present challenges.

**Growing points:**

There are approaches that lend themselves to achieving greater clarity and coherence in discussion of genome editing.

**Areas timely for developing research:**

People’s understanding should ideally be able to withstand and evolve alongside current developments in genome editing, rather than being tied firmly to specific aspects of genome editing (which may in future be supplanted).

## Why talk about genome editing?

Few topics in biology or medicine have prompted as much discussion in recent years as genome editing, the deliberate alteration of selected DNA sequences in living cells. Human genome editing has many potential therapeutic applications^1,2^ and is anticipated to be among the most important areas of biomedical innovation in the next 5 years,^3^ to say nothing of the longer-term.

Patients who could in future benefit from or otherwise be affected by genome editing need a rudimentary understanding of this subject, as do patients whose involvement is vital if genome editing research is to proceed. The latter group includes fertility patients, because any research that involves editing the genomes of human embryos will most likely be dependent on fertility patients donating surplus embryos following treatment. This was the case with recent research at the UK’s Francis Crick Institute where the genomes of human embryos were—for the first time—edited to study the function of a gene during the first few days of development.^4^

The only alternative to using surplus embryos from fertility treatment is to create embryos specifically for research. It is possible to obtain a licence to do this in the UK if supernumerary embryos are not available, but this still requires the consent of the sperm and egg donors from whose gametes the embryos will be created.^5^ Meanwhile, so-called SHEEFs (synthetic human entities with embryo-like features) are not yet sufficiently similar to embryos proper to serve as a substitute (and were they ever to become so, there may be new ethical and regulatory questions with which to contend).^6^

Even more than patients, medical practitioners in the fertility sector and more generally will increasingly require an understanding of, and clear vocabulary with which to discuss, genome editing. Fertility practitioners will be responsible for talking to patients about the option of donating embryos or gametes, for research.^7^ Practitioners in various fields will increasingly find themselves fielding patient questions about genome editing, as it progresses and as its possible uses become more widely reported (or misreported).

Finally, comprehension of genome editing and a *lingua franca* with which to discuss the subject are important prerequisites for informed debate. At present, in the UK and in many other jurisdictions, the only permitted clinical applications of human genome editing are somatic—they involve changes to the genome that will not be inherited by the next generation. In the foreseeable future, there may be compelling arguments for permitting germline genome editing in the clinic—changes to the genome that will be inherited by the next generation. Any proposed legal or regulatory change to permit this should involve thorough public debate.^8,9^

## Involving patients and practitioners

In 2016 and 2017, two UK charities—the Progress Educational Trust (PET) and Genetic Alliance UK—carried out a joint project entitled ‘Basic Understanding of Genome Editing’, in order to address the need for understanding and clear vocabulary in this area.

Two groups of participants were established for the project. One consisted of 14 fertility sector patients and practitioners, and one consisted of 18 people affected by—or caring for someone affected by—genetic disease or rare disease. Five day-long workshops were conducted with these two groups—two dedicated workshops per group, plus a larger workshop that brought the two groups together.

During these workshops and during additional online exercises, participants—whose understanding of genome editing was, in all cases, naïve at best—explored language, imagery and ideas related to the topic. They examined 2 years’ worth of media coverage of genome editing, they watched a variety of explanatory videos, they explored additional material, and they heard from—and put questions to—experts in the science and ethics of genome editing.

Most gratifyingly, towards the end of the project, these participants gave their own presentations on genome editing, drawing upon what they had learned so far. This provided invaluable insights into their understanding of the subject, revealing which concepts and terminology had proved to be either an aid or an impediment.

It became apparent that there was a palpable desire among participants for clarity and coherence in discussion of genome editing, and that certain approaches lend themselves to achieving this. The conduct and findings of the project are reported in detail elsewhere,^10^ and these findings have prompted wider discussion.^11–13^ Here, we should like to focus on some key lessons from the project and from other related developments.

## Understanding the genome

The ‘editing’ half of the term ‘genome editing’ was readily understood by most of the participants in the project, resonating as it does with the well-established metaphor of DNA as text. ‘Editing’ was taken—correctly—by most participants to mean a deliberate and specific alteration to DNA (as distinct from, say, the haphazard alterations that might result from simple exposure of an individual to a mutagenic substance or environment).

By contrast, the term ‘genome’ was not very well understood—not even by very engaged genetic disease patients, who might be expected to encounter it frequently. Participants were not confident about their grasp of this term, and several were uncertain whether it referred to something larger than or smaller than a gene. Furthermore, there was confusion about whether the genome is the same in different cells of the body.

This is not necessarily surprising, when one considers how counterintuitive use of the term ‘genome’ can be. The terms ‘gene’ and ‘genome’ originally emerged during heated debates about whether aspects of heredity were best thought of as concrete and particulate (specific molecules or portions thereof) or as more abstract and hypothetical phenomena.^14,15^

The subsequent ‘modern synthesis’ between major theories of evolution and heredity,^16^ and the discovery of the function^17^ and structure^18^ of DNA, brought the concrete and particulate meanings of the terms ‘gene’ and ‘genome’ to the fore. However, these meanings have been problematised and contested anew in the era of whole genome sequencing (and now genome editing), with latter-day definitions becoming increasingly complex and contingent.^19–21^

One way to make sense of this complex history is to say that the term ‘gene’ can refer to a tangible object (a specific portion of DNA coding for a molecule, classically for a protein) but can also refer simultaneously to an abstract concept (an inherited instruction to synthesise a molecule or to enact a function via a molecule). The dialectical tension between these two possible meanings of the term ‘gene’ is in turn manifest in the meaning of the term ‘genome’.

When botanist Hans Winkler first proposed the term ‘genome’ in 1920, he used it to mean something concrete—a haploid set of chromosomes.^22^ The term can still be used for this purpose today (provided that it is contextualised appropriately), but it can just as legitimately be used for a wide range of other purposes.

Like other biological terms with the suffix ‘-ome’, ‘genome’ connotes totality—a complete set of material. At the same time, it connotes singularity—we often talk of the genome, the definite article. However, this singularity is context-specific—a cell, an organism and a species are all entities that can be said to possess a single genome. Alternatively, these entities can be said to possess multiple genomes, provided that the distinctions between these genomes are specified (for example, distinctions between the nuclear and the mitochondrial genome, or between the germline and the somatic genome).

Once the term ‘genome’ encompasses more than a single cell (or, in the case of mitochondria, a single structure within a cell), it ceases to correspond exactly to concrete molecules and begins to acquire a more abstract and idealised character. Because DNA replication is imperfect, there is inevitably a degree of somatic mosaicism (genetic differences between cells throughout the body) in every individual.^23,24^ In recent years, it has become possible to verify this fact empirically,^25,26^ although it remains challenging to distinguish true mosaicism from anomalies attributable to imperfections in genome sequencing technology.^27^

Provided that mosaicism remains within certain boundaries, it tends to be subsumed within the concept of the individual’s genome, which is discussed as though it is replicated ubiquitously and accurately throughout most of the cells of the body. Only when drastically abnormal cells proliferate—when an individual has cancer—do we say that this individual has one or more additional ‘cancer genomes’.^28^ The more complex truth underlying usage of the term ‘genome’ is that any genome ascribed to an individual is either an idealised average of many cellular genomes (when described in theory), or a set of data from biological samples which are assumed to be representative (when sequenced in practice).

It is neither possible nor desirable to convey all of the nuances of the term ‘genome’ when discussing genome editing in public, but it is helpful nonetheless to be cognisant of and attentive to these nuances. What is most important is simply to ensure at the outset that one’s audience has some understanding of what a genome is—this cannot be taken for granted, and an explanation may well be necessary.

One way to clarify the meaning of the ‘genome’, despite the fact that it can mean different things in different contexts, is to say that it contains all of the material or information necessary for the entity to which it belongs—be it a cell, an individual or a species—to be (re)created, and for the life of that entity to be maintained. Researchers increasingly conceptualise the genome as a dynamic rather than a static object of study, but the genome can nonetheless be considered static relative to the dynamic process of its being purposefully edited by humans.

## Editing the genome

Although the meaning of the term ‘genome’ can be challengingly elusive, the corollary of this is that the term is advantageously flexible. Consequently, the term ‘genome editing’ has wide applicability, and is accurate in contexts where alternative terms might not be accurate. We therefore recommend using the term ‘genome editing’ exclusively wherever possible.

Such consistency is important, at a time when a plethora of synonyms and near-synonyms for genome editing—including ‘gene editing’, ‘genetic editing’ and ‘genomic editing’—circulate and are often used interchangeably. Participants in the ‘Basic Understanding of Genome Editing’ project were confused by this multiplicity of terms, as it was not clear to them whether the distinctions between them were distinctions without a difference. This impeded their understanding and undermined their confidence.

Helpfully, the term ‘genome editing’ can refer to an edit made to a single gene or to a part of that gene such as a single base pair,^29^ because even a change as small as this can be said to constitute a change to an entire genome to which the relevant gene belongs. Additionally, genome editing approaches including clustered regularly interspaced short palindromic repeats (CRISPR) may involve much of the genome being searched by a guide molecule in order to alight on the site of the desired edit, meaning that the editing process does in fact span the genome even if the resulting edit is minor.

At the other extreme, there are genome editing methods that can be used to delete entire chromosomes.^30,31^ The category ‘genome’ encompasses chromosomes—including, but not limited to, the genes on those chromosomes—and so chromosome deletion can also constitute genome editing, provided that a specific chromosome is purposefully deleted in a living cell.

In the same way that confusing alternatives to the term ‘genome’ currently impede understanding of genome editing, so the same is true of alternatives to the term ‘editing’. One such variant term is ‘modification’, which is liable to mislead because ‘genetic modification’ has long been associated with the introduction of transgenic (foreign) DNA into an organism—this is the common meaning of the widely used phrases ‘GM crops’ and ‘GM food’.

By contrast, editing the genome of a human or any other organism does not necessarily involve introducing any transgenic DNA. Indeed, the question of whether genome editing constitutes ‘genetic modification’ is a matter of ongoing contention, with a recent judicial opinion from the Court of Justice of the European Union suggesting that genome edited organisms could be exempt from laws governing genetically modified organisms.^32^ It is therefore advisable to avoid speaking of genetic (or gene, or genomic) ‘modification’ in relation to genome editing.

Another unhelpful alternative to the term ‘editing’ is ‘engineering’. ‘Genetic engineering’ has long been near-synonymous with ‘genetic modification’, and is therefore best avoided for the reasons given above. ‘Genome engineering’ is a slightly different case—this has become an established term of art in its own right among specialists, one whose possible meanings and methods do overlap with those of genome editing.^33^ The term is nonetheless liable to cause confusion, simply by virtue of inconsistency.

## Putting CRISPR in its place

Genome editing owes much of its current prominence to CRISPR, an approach that has transformed the field since it was pioneered in 2012.^34^ CRISPR’s advantages over earlier approaches to genome editing in terms of precision, practicability and affordability have led to it becoming a near-ubiquitous tool in genetic research, and there is now even a peer-reviewed journal dedicated solely to articles about CRISPR.^35^

Participants in the ‘Basic Understanding of Genome Editing’ project were confused by a current tendency to use the term ‘CRISPR’ as though it is a synonym for genome editing. In truth CRISPR is not a synonym but a *synecdoche* for genome editing, and a potentially misleading one at that. The approaches that preceded CRISPR are not obsolete, but remain important in the present day (notwithstanding their limitations).

For example, transcription activator-like effector nucleases (TALENs) is an earlier genome editing approach that has been used recently in the UK to reverse advanced leukaemia in two infants, an achievement that has been widely reported and lauded.^36^ Another earlier approach, zinc finger nucleases (ZFNs), has been used recently in a clinical trial in the USA—to treat Hunter syndrome—where genome editing components were, for the first time, introduced directly into a patient’s body (rather than being used on cells or tissues removed from a patient or donor’s body and then reintroduced into the patient).^37^

CRISPR was not the first word in genome editing, nor is it likely to be the last. At one juncture, it seemed that the successor to CRISPR might be an approach named NgAgo (*Natronobacterium gregoryi* Argonaute), but the paper where this was proposed was eventually retracted when other researchers were unable to replicate its findings.^38^ Instead, a more plausible successor to CRISPR has emerged in the form of ‘base editing’^39^, which has already been used in research to edit the genomes of human embryos.^40^

If ‘CRISPR’ is a popular but misleading synecdoche for genome editing, then this is even more true of ‘CRISPR/Cas9’. Cas9 (CRISPR associated protein 9) is the nuclease (the ‘cutting’ molecule) most commonly used in CRISPR genome editing, but work is ongoing with alternative nucleases (such as Cpf1^41^) and it is not inconceivable that Cas9 could be supplanted as the nuclease of choice in future. A current tendency to append the term ‘Cas9’ to the term ‘CRISPR’ in public discussion by default, rather than doing so only when there is a reason to be this specific, narrows unnecessarily the technology that is being discussed.

Just as there are examples of genome editing that are not CRISPR, so there are examples of CRISPR that are not genome editing. In fact, CRISPR research predates genome editing research by a number of years—the term ‘CRISPR’ originally refers to a naturally occurring phenomenon, first observed in bacteria in 1987,^42^ which defends these bacteria against invading viruses. It is this natural phenomenon that has been adapted into an ingenious approach to genome editing.

More recently, researchers have created a real-time film of a CRISPR process at the molecular scale.^43^ The paper where this was described has proved popular,^44^ but also makes it clear that in order to create the film, researchers had to contrive a situation which took CRISPR outside the auspices of genome editing—rather than alter DNA sequences in living cells, they altered sequences within fragments of purified DNA attached to a surface. The resulting film documented only part of the ‘editing’ process—DNA was cut, but was not then repaired—which further disqualifies this example of CRISPR from being considered ‘genome editing’.

A final distinction that can give rise to confusion is that not all CRISPR editing is necessarily *genome* editing. CRISPR (and other approaches) can also be used for *epigenome* editing, and this too has potential therapeutic applications.^45,46^ The term ‘epigenome’ is as challenging to define as the term ‘genome’ (if not more so), and we will not attempt to do so here—suffice to say that ‘editing’ epigenomes involves altering the pattern of gene expression in living cells, *without* making any alterations to the DNA sequences in those cells.

## Conclusions

The story of genome editing is not complete but rather is still unfolding and is liable to take unexpected turns. Seemingly promising avenues may transpire to be *culs-de-sac*, while areas which seem arcane and unimportant may transpire to be tremendously significant. Patients and practitioners need to be furnished with an understanding of genome editing that can withstand and evolve alongside these sorts of developments, rather than being tied firmly to CRISPR (still less CRISPR/Cas9).

When discussing genome editing in public, attention should be given in the first instance not to CRISPR, but instead to addressing the following three questions in the following order of priority.

- What is a genome?
- What is genome editing?
- What can genome editing be used for?

It is important not just to prioritise but to reiterate one’s explanations. Participants in the ‘Basic Understanding of Genome Editing’ project found it challenging to retain new information—even after hearing a thorough and well-received explanation during a workshop, they sometimes struggled to recall important points from this explanation when they attended a subsequent workshop.

Opportunities should therefore be sought not just to offer but to repeat and reinforce explanations of genome editing, so that public and professional understanding of this increasingly important field can endure and grow.

## Biography

Sandy Starr is Communications Manager at the Progress Educational Trust (PET), a charity which improves choices for people affected by infertility and genetic conditions where he has worked for more than 10 years. He writes for a wide range of publications, most recently *Microbiology Today* and *Health Expectations*. He also serves on the Ethics Advisory Board of the world’s largest autism research project, *European Autism Interventions: A Multicentre Study for Developing New Medications*. In 2017, he represented PET in an address to the Science and Technology Select Committee of the House of Commons, on the subject of human embryo research.
